# Use of a DVD to provide dietary and lifestyle information to pregnant women who are overweight or obese: a nested randomised trial

**DOI:** 10.1186/s12884-014-0409-8

**Published:** 2014-12-12

**Authors:** Malgorzata A Szmeja, Courtney Cramp, Rosalie M Grivell, Andrea R Deussen, Lisa N Yelland, Jodie M Dodd

**Affiliations:** The University of Adelaide, Discipline of Obstetrics & Gynaecology and The Robinson Research Institute, Women’s & Children’s Hospital, 72 King William Road, North Adelaide, South Australia 5006 Australia; Department of Perinatal Medicine, Women’s and Children’s Hospital, North Adelaide, South Australia Australia; Women’s and Children’s Health Research Institute, North Adelaide, South Australia Australia; The University of Adelaide, School of Population Health, Adelaide, South Australia Australia

**Keywords:** Obesity, Pregnancy, Randomised trial, Evaluation of information provision

## Abstract

**Background:**

We conducted a nested randomised trial to evaluate the effect of an educational DVD, providing information about healthy food choices and exercise during pregnancy, on diet and physical activity, among pregnant women who were overweight or obese.

**Methods:**

We conducted a nested randomised trial within the context of the LIMIT randomised trial. Women were eligible with a singleton pregnancy between 10 and 20 weeks gestation, and body mass index at the time of their first antenatal appointment of ≥25 kg/m^2^. All women who were randomised to the Lifestyle Advice Group of the LIMIT trial received a series of consultations with both research dieticians and research assistants, in addition to standard written dietary and exercise materials (Standard Materials Group). Women randomised to the DVD Group received the same consultations and written materials, and additionally received an educational DVD (DVD Group). The primary study outcome was the Healthy Eating Index. Other study outcomes included physical activity, and gestational weight gain. Women completed a qualitative evaluation of all the materials provided.

**Results:**

1,108 women in the LIMIT Lifestyle Advice Group participated in the nested trial, with 543 women randomised to the DVD Group, and 565 women to the Standard Materials Group. Women who received the DVD compared with those who did not, had a higher mean Healthy Eating Index at 36 weeks gestation (73.6 vs 72.3; adjusted mean difference 1.2; 95% CI 0.2 to 2.3; p = 0.02), but not at 28 weeks gestation (73.2 vs 73.5; adjusted mean difference −0.1; 95% CI −1.1 to 0.9; p = 0.82). There were no statistically significant differences in physical activity or total gestational weight gain. While most women evaluated the materials positively, frequency of utilisation was poor.

**Conclusions:**

Ongoing attention to the delivery of information is required, particularly with the increased use and availability of digital and multi-media interactive technologies.

**Trial registration:**

Australian and New Zealand Clinical Trials Registry ACTRN12607000161426

## Background

Overweight (defined as a body mass index (BMI) between 25.0 and 29.9 kg/m^2^) and obesity (defined as a BMI greater than or equal to 30.0 kg/m^2^) [1] are associated with significant health complications for women during pregnancy and childbirth, with a well documented increase in the risk of adverse outcomes, both for women and their infants [2-4]. It is estimated that approximately 35% of pregnant women in Australia have a BMI above 25 kg/m^2^ [3]. More recent population based data would indicate that this is approaching 50% of pregnant women [5], figures that are consistent with those from the United States [6,7].

While there are an increasing number of reports in the literature outlining the association between high maternal BMI and pregnancy complications, more limited information is available describing the impact of antenatal dietary and lifestyle interventions for women who are overweight or obese. Furthermore, there is increasing recognition that the method of information provision is important in increasing the transfer of knowledge and information retention [8]. This may be of considerable importance in the setting of dietary information presented for weight management. A number of randomised studies have evaluated the use of educational material presented via a DVD or video format to enhance knowledge and treatment compliance, for self management of type 2 diabetes and treatment compliance for parents of children prescribed antibiotics, women choosing mode of birth after prior caesarean birth and for couples undergoing embryo transfer [9-12]. While the clinical care setting of each of these studies varied considerably, and different measures of compliance or success were utilised, the conclusion was that the addition of the DVD or video improved treatment compliance and was an effective method of delivering health care information [9-12]. It is unclear if these findings are applicable to weight management in adult populations, particularly for women during pregnancy.

The aims of the current study, conducted in the context of the LIMIT randomised trial, were two-fold. Firstly, we evaluated in a nested randomised trial, the effect of an educational DVD as a tool to provide information about healthy food choices and exercise during pregnancy on diet and physical activity outcomes. Secondly, we evaluated written materials provided to pregnant women who were overweight or obese, and whether use of these materials improved diet and physical activity outcomes, as part of an antenatal dietary intervention trial.

## Methods

### Study design

We conducted a nested randomised trial in the context of the LIMIT randomised trial, the protocol [13], and the effect of the antenatal lifestyle intervention for women who are overweight or obese on maternal and infant health outcomes having been reported previously [14-17].

### Inclusion and exclusion criteria

Women were eligible for inclusion in the nested randomised trial who had been randomised to the Lifestyle Advice Group of the LIMIT trial [17]. Briefly, women identified with a BMI ≥25 kg/m^2^ and live singleton pregnancy between 10^+0^ and 20^+0^ weeks’ gestation at the time of their first antenatal visit were eligible to participate.

### Trial entry

At the time of a woman’s first antenatal appointment, her height and weight were obtained and BMI calculated. All women provided written informed consent to participate.

### Randomisation, blinding and masking

Randomisation occurred by telephoning the central randomisation service, which utilised a computer-generated schedule, with balanced variable blocks. During the period of the LIMIT randomised trial, we conducted a nested randomised trial, in which women who were randomised to the ‘Lifestyle Advice’ group underwent further randomisation to the DVD Group or to the Standard Materials Group.

### Treatment schedules

#### DVD Group

Women who were randomised to the DVD Group received the standard written materials and series of consultations as described subsequently, in addition to the informational DVD. The informational DVD was a specifically prepared educational tool containing information about healthy eating during pregnancy, serving sizes, and exercise during pregnancy. The information content of the DVD was the same as that presented to women in the intervention sessions and written materials.

#### Standard materials group

Women randomised to the standard materials group received the standard written materials and series of consultations as described subsequently.

#### Both treatment groups

All women who participated in the nested randomised trial and the evaluation of the dietary materials, were randomised to receive the lifestyle intervention, and were provided with dietary advice consistent with current Australian standards [18]. Specifically, women received individualised advice to maintain a balance of carbohydrates, fat and protein, to reduce intake of foods high in refined carbohydrates and saturated fats, while increasing intake of fibre, and promoting consumption of two servings of fruit, five servings of vegetables, and three servings of dairy each day [18]. Physical activity advice primarily encouraged women to increase their amount of walking and incidental activity [19].

Within two weeks of randomisation, women attended a planning session with a research dietician, during which a detailed dietary and exercise history was obtained. Women were provided with individualised information, including meal plans, healthy recipes that were quick to prepare, simple food substitutions (including reducing sugar-sweetened soft drinks and fruit juices, reducing added sugar and foods high in refined carbohydrates, and introducing low-fat alternatives), healthy snack and eating out options, and guidelines for healthy food preparation. Women were encouraged to set achievable goals for dietary and exercise change, and were supported to make these lifestyle changes and to self-monitor their progress through the use of a work book provided and ongoing contact with research staff. Women were encouraged to identify potential barriers to them implementing their dietary and physical activity goals. Using these perceived barriers, women were assisted to problem solve, and to develop individualised strategies to facilitate their successful implementation.

All women received this information in both verbal and written formats (comprising the “Nutrition in Pregnancy”, “Exercise in Pregnancy”, and “Pregnancy Record” books), which was reinforced during subsequent inputs provided by the research dietician (at 28 weeks gestation) and trained research assistants (via telephone call at 22, 24, and 32 weeks gestation and a face-face visit at 36 weeks gestation). The “Nutrition in Pregnancy” book contained written and pictorial information including common pregnancy complications that may be associated with high degrees of gestational weight gain, healthy eating during pregnancy, food groups with specific examples, recommended number of servings per day, portion sizes (including ‘rate your plate’), and food label reading. The “Exercise in Pregnancy” book provided information about the benefits of physical activity during pregnancy, practical tips to increase physical activity, as well as safety information. The “Pregnancy Record” provided women with a workbook tool in which to record their dietary and exercise goals, and to self-monitor their progress. The “Healthy Cooking” book provided women with sample weekly menu plans and healthy recipe ideas.

For all women participating in this study, the remainder of their pregnancy care was according to the practices of their caregiver and the local hospital guidelines where they planned to birth.

### Study endpoints

The primary study outcome was the woman’s Healthy Eating Index (HEI) during pregnancy, as determined by self-completed food frequency questionnaire.

Women completed the Harvard Semi-quantitative Food Frequency questionnaire (The Willett Questionnaire) at 28 and 36 weeks gestational age. The Willett questionnaire was developed in 1985 in the United States to measure the daily intake of nutrients from 126 food items, with an indication of standard portion size, divided into seven food groups [20], and has been validated for use during pregnancy [21], and in an Australian context [22]. Responses to this questionnaire were considered invalid and hence excluded if more than 25% of responses were missing, or if total energy intake was unrealistic (<4500 kJ or >20000 kJ) [23]. Using information derived from the food frequency questionnaire, the 2005 HEI was used as an index of diet quality [24], consisting 12 components, with a maximum score of 100. Total fruit (including 100% juice), whole fruits (excluding juice), total vegetables, dark green and orange vegetables, vegetables and legumes (legumes included as a vegetable only after the Meat and Beans standard was met), total grains and whole grains categories are each scored out of five. Milk (all products made from cow’s milk, goat’s milk and soy beverages but excluding products that are primarily fat such as butter, cream, sour cream and cream cheese), meat and beans (meat products, eggs, nuts, seeds, soy-based products and legumes), oils (fats that are liquid at room temperature, from a plant source and not described as ‘hydrogenated’ or ‘shortening’ including oils from plant, fish, nuts and seeds or margarines), saturated fat and sodium are each scored out of 10. Calories derived from solid fats (all excess fat from the milk, meat and beans components beyond that would be consumed if only the lowest fat forms were eaten, solid fats added to foods in preparation or at the table including cream, butter, stick margarine, regular or low-fat cream cheese, lard, meat drippings, cocoa and chocolate), alcoholic beverages and added sugars (SoFAAS) are scored out of 20. Scores for saturated fat, sodium and calories derived from solid fats, alcohol and added sugars are reverse scored, where a higher score indicates lower consumption. A HEI above 80 is considered good, between 50–80 needs improvement and below 50 is considered poor. The HEI has been validated for use in a pregnant population [25].

Women completed the Short Questionnaire to Assess Health-enhancing physical activity (SQUASH) [26], also at 28 and 36 weeks gestation. The 11-item questionnaire evaluates the time spent in different categories of physical activity, including commuting, leisure, household and incidental, and work related activities. Responses to this questionnaire were considered invalid and hence excluded if the total hours of activity reported per week exceeded the number of hours in a week. Each activity was assigned an estimate of intensity in Metabolic Equivalent Task units (METs) [27]. As the SQUASH questionnaire reports physical activity during an average week, MET-minutes per week (METs/wk) were calculated as duration (in minutes) × frequency (days per week) × MET intensity.

Total gestational weight gain was calculated as the difference between measured weight at 36 weeks gestation and that obtained from the antenatal booking visit. Average weekly gestational weight gain was calculated by dividing total weight gain by the time between measurements in weeks, to account for differences in timing of measurements obtained at trial entry and 36 weeks gestation.

All women were asked to self-complete a questionnaire at four months post-partum, in which they were asked to indicate whether they agreed or disagreed with a series of statements related to the information presented during the course of pregnancy, including whether it was easy to understand, whether they considered it useful, whether the information presented assisted in making healthier food choices, and how often they referred to the materials. Women were also asked to identify whether their knowledge of healthy food choices and exercise during pregnancy had improved. For each question there were five alternate answers ranging from “strongly agree” to “strongly disagree”, or when referring to the frequency of use from “never” to “more than once every week”.

### Sample size

The primary endpoint was the Healthy Eating Index score at 28 and 36 weeks gestation. Sample size calculations for the LIMIT randomised trial indicated 1090 women were required in the Lifestyle Advice Group [13]. Assuming a mean (SD) Healthy Eating Index score during pregnancy of 72.4 (7.1) [14], and accounting for a response rate of 65%, this sample size provides 90% power to detect a difference in mean Healthy Eating Index of 2 points between the DVD Group and the Standard Materials Group (80% power, 2-sided alpha = 0.025 to correct for multiple comparisons due to outcome assessment at 28 and 36 weeks).

### Analysis and reporting

Analyses were performed on an intention to treat basis, according to the treatment group allocated at randomisation. Continuous outcomes were analysed using linear regression models, with treatment effects expressed as differences in means. Binary outcomes were analysed using log binomial regression models, with treatment effects expressed as relative risks. Both unadjusted and adjusted analyses were performed, with adjustment for the stratification variables centre, parity and BMI, as well as age, socio-economic status and smoking status. For the Healthy Eating Index and total physical activity, a p value of <0.025 was required for statistical significance to correct for multiple comparisons due to the two assessment time points at 28 and 36 weeks gestation. For other outcomes measured at a single time point, a p value <0.05 was used to indicate significance and no adjustment was made for multiple comparisons due to the multiple comparisons considered. All analyses were performed based on the available data using SAS v9.3 (Cary, NC, USA).

Responses to the questionnaire assessing women’s opinions on the written materials and DVD were assessed descriptively. To determine whether use of the relevant materials was associated with the Healthy Eating Index or total physical activity at 36 weeks gestation, women were classified as users if they indicated they referred to the materials occasionally, sometimes, or often during pregnancy, and non-users if they referred to the materials rarely or never. Mean scores were compared between users and non-users using linear regression models with and without adjustment for centre, parity, BMI, age, socio-economic status, and smoking status.

### Ethics

Ethics approval was granted by the Women’s and Children’s Local Health Network Human Research and Ethics Committee at the Women’s and Children’s Hospital, the Central Northern Adelaide Health Service Ethics of Human Research Committee (Lyell McEwin Hospital) and the Flinders Clinical Research Ethics Committee (Flinders Medical Centre).

## Results

**Figure 1:**
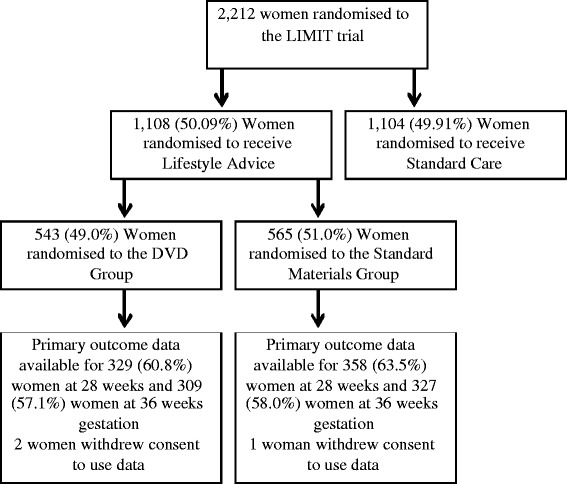
**Flow of participants in the nested randomised trial.**

Of the 1108 women randomised the LIMIT Lifestyle Advice Group [17], 543 (49.0%) were randomised to the DVD Group, and 565 (51%) to the Standard Materials Group. The flow of participants is outlined in Figure 1, and baseline characteristics of women at the time of trial entry are presented in Table 1. Maternal demographics were similar between the two treatment groups.

**Table 1:** **Baseline characteristics of randomised participants**

***Characteristic***	***DVD Group (N = 541)***	***Standard Materials Group (N = 564)***
Maternal Age (Years)^*^	29.2 (5.6)	29.4 (5.3)
Gestational Age at Entry (Weeks)^+^	14.0 (11.9-17.3)	14.1 (12.0-16.7)
Body Mass Index (kg/m^2^)^+^	31.0 (28.0-35.9)	31.1 (28.1-35.8)
Body Mass Index Category^#^		
BMI 25·0-29·9	225 (41.6)	233 (41.3)
BMI 30·0-34·9	156 (28.8)	170 (30.1)
BMI 35·0-39·9	94 (17.4)	108 (19.1)
BMI > = 40·0	66 (12.2)	53 (9.4)
Public Patient^#^	530 (98.0)	551 (97.7)
Weight (kg)^*^	89.2 (18.2)	88.0 (16.3)
Height (cm)^*^	165.1 (6.7)	164.6 (6.5)
Caucasian^#^	488 (90.2)	507 (89.9)
Smoker^#^	74 (13.7)	80 (14.2)
Nulliparous^#^	224 (41.4)	233 (41.3)
Index of Socio-economic Disadvantage^		
Unknown	1 (0.2)	1 (0.2)
Quintile 1 (Most Disadvantaged)	183 (33.8)	157 (27.8)
Quintile 2	128 (23.7)	143 (25.4)
Quintile 3	68 (12.6)	105 (18.6)
Quintile 4	74 (13.7)	76 (13.5)
Quintile 5 (Least Disadvantaged)	87 (16.1)	82 (14.5)

Includes all women randomised who did not withdraw consent to use their data.

*mean and standard deviation.

^+^median and interquartile range.

^#^number and %.

^Socioeconomic index as measured by SEIFA.

### Maternal healthy eating index and physical activity

Valid response rates for the food frequency questionnaire and physical activity questionnaire were similar between treatment groups (Table 2). At 28 weeks gestation there was no evidence of a difference in the Healthy Eating Index of women who did or did not receive the DVD (adjusted mean difference (AMD) -0.12, 95% confidence interval (CI) -1.10 to 0.87, p = 0.82). However, women who received the DVD reported higher mean HEI scores at 36 weeks compared with women who received written materials alone (AMD 1.20, 95% CI 0.16 to 2.25, p = 0.02). There were no statistically significant differences identified in total physical activity at either 28 or 36 weeks gestation, or total or average gestational weight gain between women who received the DVD, and those who received standard written materials alone (Table 3).

**Table 2:** **Response rates by treatment group**

***Questionnaire***	***DVD Group (N = 541)***	***Standard Materials Group (N = 564)***
Food Frequency Questionnaire*		
28 weeks	329 (60.8)	358 (63.5)
36 weeks	309 (57.1)	327 (58.0)
Physical Activity Questionnaire*		
28 weeks	350 (64.7)	376 (66.7)
36 weeks	333 (61.6)	359 (63.7)
Knowledge Questionnaire	287 (53.0)	302 (53.5)
Evaluation of Written Materials	276 (51.0)	292 (51.8)
Evaluation of DVD	152 (28.1)	N/A

Values are number (%).

*Valid questionnaire responses.

**Table 3:** **Study outcomes by treatment group**

***Outcome***	***DVD Group (N = 541)***	***Standard Materials Group (N = 564)***	***Unadjusted Treatment Effect (95% CI)***	***Unadjusted P-value***	***Adjusted Treatment Effect (95% CI)***	***Adjusted P-value***
Healthy Eating Index Score						
28 weeks	73.22 (6.79)	73.47 (6.45)	−0.26 (−1.24, 0.73)	0.61	−0.12 (−1.10, 0.87)	0.82
36 weeks	73.60 (6.58)	72.34 (7.00)	1.26 (0.20, 2.31)	0.02	1.20 (0.16, 2.25)	0.02
Physical Activity						
28 weeks	7040.20 (3968.84)	6967.30 (3943.89)	72.90 (−502.19, 648.00)	0.80	121.48 (−453.94, 696.90)	0.68
36 weeks	5756.54 (4062.39)	5865.81 (3858.77)	−109.27 (−698.64, 480.09)	0.72	−125.57 (−717.73, 466.59)	0.68
Total Gestational Weight Gain (kg)	9.09 (5.75)	9.66 (5.73)	−0.58 (−1.33, 0.18)	0.13	−0.58 (−1.30, 0.13)	0.11
Average Weekly Gestational Weight Gain (kg)	0.43 (0.29)	0.46 (0.28)	−0.02 (−0.06, 0.01)	0.23	−0.02 (−0.06, 0.01)	0.19
Increased knowledge of healthy food choices^*^	201/285 (70.53%)	203/301 (67.44%)	1.05 (0.94, 1.17)	0.42	1.06 (0.95, 1.18)	0.30
Increased knowledge of exercise^*^	224/285 (78.60%)	220/301 (73.09%)	1.08 (0.98, 1.18)	0.12	1.05 (0.96, 1.16)	0.28
Improved health during pregnancy^*^	229/285 (80.35%)	228/301 (75.75%)	1.06 (0.88, 1.27)	0.53	1.05 (0.96, 1.14)	0.28

Values are mean (SD) and treatment effects are differences in means unless otherwise indicated.

^*^Values are number (%) and treatment effects are relative risks.

### Post-partum assessment of maternal self-reported knowledge

Knowledge questionnaires were returned by 589 women, with a response rate of around 53% in each group (Table 2). There were no statistically significant differences identified between women in the DVD group and those who received standard written materials in their self-reported knowledge of healthy food choices (DVD Group 201/285 (70.5%) versus Standard Materials Group 203/301 (67.4%), adjusted relative risk (ARR) 1.06, 95% CI 0.95 to 1.18; p = 0.30), or exercise during pregnancy (DVD Group 224/285 (78.6%) versus Standard Materials Group 220/301 (73.1%), ARR 1.05, 95% CI 0.96 to 1.16; p = 0.28) (Table 3). Similarly, there were no statistically significant differences identified between the two groups in women’s sense of improved health (Table 3).

### Qualitative assessment of the provided study materials

A total of 568 women provided written feedback in relation to the provided written study materials and response rates were similar between groups (Table 2). While the majority of women both “liked” the nutrition in pregnancy book (78.3%), and found the information easy to follow (87.3%), relatively few women referred to the book sometimes or often during pregnancy (21.3%) or in the postpartum period (9.9%). There was no evidence to suggest that use of the nutrition pregnancy book during pregnancy influenced the Healthy Eating Index (Table 4). Similarly, the majority of women (67.3%) “liked” the exercise in pregnancy book, and found the information easy to follow (78.2%), although few women referred to the book sometimes or often during pregnancy (15.0%), or in the postpartum period (5.5%). Use of the exercise in pregnancy book was not associated with an improvement in the total physical activity (Table 4).

**Table 4:** **Effect of book and DVD use on diet and exercise outcomes at 36 weeks gestation**

***Outcome***	***Information Source***	***Users***	***Non-Users***	***Unadjusted Effect (95% CI)***	***Unadjusted P-value***	***Adjusted Effect (95% CI)***	***Adjusted P-value***
HEI	Nutrition in Pregnancy Book	73.76 (7.16)	73.21 (6.74)	0.55 (−0.72, 1.82)	0.39	0.60 (−0.66, 1.86)	0.35
Total Activity Score	Exercise and Pregnancy Book	5758.30 (3686.65)	5827.75 (3858.91)	−69.45 (−790.51, 651.61)	0.85	−2.45 (−729.66, 724.76)	0.99
HEI	DVD	73.76 (7.11)	75.60 (6.41)	−1.83 (−4.93, 1.26)	0.25	−1.61 (−4.59, 1.36)	0.29
Total Activity Score	DVD	6449.69 (3722.84)	6224.12 (4430.09)	225.57 (−1780.00, 2231.14)	0.83	82.66 (−1924.09, 2089.42)	0.94

Values are mean (SD) and effects are differences in means for users of the information source compared with non-users.

A total of 152 (28.1%) women randomised to the DVD Group evaluated the material presented in the DVD, of whom 59 (33.8%) were overweight and 93 (61.2%) were obese. Eighty-eight women (57.9%) agreed or strongly agreed that overall the information presented was easy to understand, as was the information presented about food groups and serving sizes. Seventy-eight women (51.3%) agreed or strongly agreed that overall the DVD was useful. Eighty-five women (55.9%) agreed or strongly agreed that they liked the DVD, and 70 women (46.1%) agreed or strongly agreed that the DVD helped in making healthier food choices. Following the initial viewing of the DVD, 51.3% of women referred to it at least once again during pregnancy, although only 22.4% of women used the information contained in the DVD following the birth of their baby, and this was similar for overweight and obese women. DVD use was not associated with a change in either the Healthy Eating Index or the total physical activity (Table 4).

## Discussion

The results of this nested randomised trial indicate that overweight and obese women reported high rates of knowledge of healthy food choices and exercise during pregnancy. The addition of an educational DVD was associated with modest improvements in maternal diet as measured by the Healthy Eating Index at 36 weeks gestation, but was no more effective in improving physical activity than the information presented in standard consultations and the written study materials. The information presented in the DVD was evaluated by women in a positive manner, the majority either agreeing or strongly agreeing that the information was easy to understand and assisted in making healthier food choices. Similarly, the written materials were viewed positively by participants, although frequency of use was low, and did not correlate with changes in the Healthy Eating Index, physical activity score, or gestational weight gain.

A potential limitation of our study is the reliance on maternal recall with regards to frequency of use, not only of the written study materials, but also of the DVD provided. While this recall may be subject to bias with women responding four months after birth, it would be anticipated that the bias would be operating evenly across both treatment groups, particularly given that the baseline characteristics of participants were comparable at the time of trial entry. Furthermore, we identified no correlation between reported frequency of use of the materials, including the DVD, and changes in reported dietary intake or physical activity, both of which were assessed at two time points during pregnancy. Additionally, there was no correlation between reported frequency of use of the materials and more objective measures of total and average weekly gestational weight gain. A further limitation is the overall low response rate, which may introduce potential non-response bias, with those women who responded potentially more likely to view the materials in a positive fashion. As highlighted previously [17], the majority of women participating in the LIMIT randomised trial were of white Caucasian ethnicity, and our findings may therefore not be generalizable to other pregnant populations.

We have previously reported findings of improved maternal diet quality, including increased consumption of fruits and vegetables and a reduction in the percentage of energy obtained from saturated fats, among overweight and obese pregnant women who received an antenatal dietary and lifestyle intervention [14,17]. Furthermore, women who received the intervention, significantly increased their physical activity, equivalent to 15–20 minutes brisk walking on most days of the week [14]. These relatively modest changes in maternal diet and physical activity have been associated with a significant 18% relative risk reduction in the chance of infant birth weight above 4.0 kg [17] and 4.5 kg [15].

While provision of the DVD in this nested randomised trial was associated with modest improvement in the Healthy Eating Index at 36 weeks gestation only, there were no differences identified in physical activity score, and the findings could, therefore, represent a chance occurrence. When compared with women of normal BMI, women who are overweight or obese demonstrate poorer diet quality during pregnancy [28], which continues into the early postpartum period [29], consisting specifically of a reduction in dietary intake of grains, vegetables, iron, and folate [28,30,31]. While poor diet quality during pregnancy, as measured by the HEI, has been associated with an increased risk of adverse pregnancy outcomes, including glucose intolerance and pre-eclampsia [32], the relative impact of the quite modest differences observed in this nested randomised trial remains uncertain. In non-pregnant adult populations, a decrease in dietary quality using a variety of measures of dietary intake has been associated with increased weight gain over time [33], and increased risk of mortality and morbidity, specifically in relation to cardiovascular disease [34]. While some studies report subtle differences in the Healthy Eating Index and improvements in health and reduced complications of type 2 diabetes [35], other studies report larger differences in diet quality, in association with changes in blood pressure and other measures of cardiometabolic disease, including cholesterol [36,37].

A number of randomised trials in different clinical settings and populations have reported that the provision of audio-visual information in addition to that provided in a standard consultation enhances patient knowledge and understanding [9-12]. King and colleagues [9] evaluated an educational DVD and booklet in addition to the information provided in a standard consultation on the degree of post-prandial glucose control among individuals with type 2 diabetes. In this study, provision of additional audio-visual information was associated with a significant increase in reported self-monitoring of blood glucose concentrations, a reduction in post-prandial hyperglycaemia, and a lower mean blood glucose concentration, when compared with standard care [9].

Taylor and colleagues [10] evaluated the effectiveness of the addition of audio-visual information to a standard written leaflet in improving parental knowledge and attitudes about the judicious use of antibiotics in children. At assessment six weeks after the intervention, parents who received the audio-visual presentation were more likely to indicate knowledge change and modification of attitudes, favouring the judicious use of antibiotic therapy in their child [10].

There have been several studies that have evaluated the use of an audio-visual tool in clinical scenarios related to obstetrics and gynaecology [11,12]. Farnworth and colleagues [11] developed an audio-visual tool to assist women choosing mode of birth following a prior caesarean section. A total of 32 women were recruited to the study, of whom 16 were allocated to receive the intervention [11]. Although the intervention was associated with a reduction in decisional conflict scores representing uncertainty about their mode of birth, the differences were not statistically significant when compared with routine information provision [11]. While qualitative analysis of the intervention suggested that women experienced an improved ability to reach a decision about their mode of birth, most women who received the intervention did not consider it more useful than the standard information brochure provided by the hospital [11].

The trial conducted by Hope [12] involved 131 couples presenting for infertility treatment, to evaluate the effectiveness of an educational DVD in influencing attitudes and increasing the acceptability of elective single embryo transfer. While women in both groups reported improved knowledge, those who received the DVD were more aware of potential risks and complications associated with a multiple pregnancy [12]. Following the delivery of the intervention, couples who received the DVD were significantly more likely to indicate a preference for elective single embryo transfer when compared with couples who received the standard information brochure (DVD group: 76/92 (82.6%) versus Standard brochure group: 68/102 (66.7%), p = 0.014).

The results of these randomised trials, while addressing different research questions, and utilising different methods of evaluation, all indicated that the use of an informational DVD was associated with improved knowledge and compliance. However, there is more limited information available about the role of audio-visual tools in the context of weight management during pregnancy.

We have identified a single study addressing the role of a CD-ROM as a tool to provide information to pregnant and post-partum women with regards to exercise [38]. Fifty women were recruited to the study (25 pregnant and 25 post-partum), and while the study was stated to be randomised, there was significant imbalance in the allocation to treatment groups (40 women in the intervention group versus 10 women in the control group) [38], raising concerns about the process of randomisation and integrity of the trial processes. While the study reported that the provision of the intervention CD-ROM was associated with improved exercise knowledge and self-efficacy during pregnancy and the postpartum period [38], the results should be interpreted with caution given the identified methodological flaws.

The evaluation of study materials from the LIMIT randomised trial indicate that the information was presented in a format that women found both easy to follow and useful, although frequency of utilisation was poor. It is recognised that the simple provision of information, either written or visual, is insufficient to initiate behaviour modification resulting in changes in dietary intake or physical activity [8]. Many consider pregnancy to be a “teachable moment” [39], when women are potentially receptive, and positive towards opportunities to improve not only their own health and wellbeing, but that of their unborn baby [40]. However, many women consider information provided by health professionals with regards to diet and weight gain during pregnancy, to be both contradictory and inadequate [41,42], with the advice a woman receives from family members considered to be more influential [42]. Findings from our group indicate that while pregnant women who are overweight or obese acknowledge the benefits of healthy eating, most report limited self efficacy to initiate behaviour change [43], prompting the need to further evaluate not only the effect of individual psychological characteristics in achieving successful changes [44], but also the way that information and resources are provided to women.

Smart-phone applications and mobile phone technologies are being utilised increasingly, not only as diagnostic tools for clinicians, but also as self-monitoring tools for individual patients [45-48], although the validity of the scientific information presented has been questioned, particularly in relation to those applications used by consumers [49]. Smart-phone applications have been used in adult weight loss settings, often as an adjunct to standard consultations, particularly as a means of facilitating access, increasing participant engagement, and as a tool to overcome potential barriers associated with traditional face-to-face health care interactions [50]. A recent systematic review of randomised trials evaluating smart-phone and mobile applications to facilitate a change in health among women [51] identified a number of studies comparing text messaging in combination with standard consultations, with standard consultations alone to facilitate weight loss [52-60]. The majority of trials reported an increase in weight loss and improvement in other biochemical measures among individuals who received additional text-messaging support [52-60], with those applications facilitating self-monitoring of progress, goal setting and behavioural feedback among the most highly rated by participants [61].

Smart-phone and mobile technology use is increasing in pregnancy care settings [62], and while more than 1500 applications [63] are available to the public, rigorous evaluation of both content and efficacy has been limited. We are aware of three randomised trials in a pregnancy setting evaluating the use of SMS or text-messaging support to improve pregnancy wellbeing [64,65] and target quit smoking [66]. While this additional support has been reported to improve maternal satisfaction and reduce anxiety during pregnancy [64,65], the role of mobile technology in facilitating change in dietary intake and physical activity patterns, particularly among pregnant women who are overweight or obese, remains to be determined.

## Conclusion

The findings of our nested randomised trial indicate that the provision of an informational DVD was associated with an improvement in dietary quality at 36 weeks gestation, but was not associated with improvements in physical activity or gestational weight gain. While most women evaluated the materials positively, frequency of utilisation was poor. Ongoing attention to the structure, delivery, and robust evaluation of antenatal dietary and lifestyle interventions, including the method of information provision, is required, particularly in an era characterised by increased use and availability of digital and multi-media interactive technologies.
